# Adipose stem cells can secrete angiogenic factors that inhibit hyaline cartilage regeneration

**DOI:** 10.1186/scrt126

**Published:** 2012-08-24

**Authors:** Christopher SD Lee, Olivia A Burnsed, Vineeth Raghuram, Jonathan Kalisvaart, Barbara D Boyan, Zvi Schwartz

**Affiliations:** 1 Wallace H. Coulter Department of Biomedical Engineering and Institute for Bioengineering and Bioscience, Georgia Institute of Technology, 315 Ferst Drive NW, Atlanta, GA, 30332-0363, USA; 2 Georgia Pediatric Urology, 5445 Meridian Marks Rd NE #220, Atlanta, GA, 30342-1341, USA; 3 Department of Periodontics, University of Texas Health Science Center at San Antonio, 7703 Floyd Curl Drive, San Antonio, TX, 78229-3900, USA

## Abstract

**Introduction:**

Adipose stem cells (ASCs) secrete many trophic factors that can stimulate tissue repair, including angiogenic factors, but little is known about how ASCs and their secreted factors influence cartilage regeneration. Therefore, the aim of this study was to determine the effects ASC-secreted factors have in repairing chondral defects.

**Methods:**

ASCs isolated from male Sprague Dawley rats were cultured in monolayer or alginate microbeads supplemented with growth (GM) or chondrogenic medium (CM). Subsequent co-culture, conditioned media, and in vivo cartilage defect studies were performed.

**Results:**

ASC monolayers and microbeads cultured in CM had decreased FGF-2 gene expression and VEGF-A secretion compared to ASCs cultured in GM. Chondrocytes co-cultured with GM-cultured ASCs for 7 days had decreased mRNAs for *col2*, *comp*, and *runx2*. Chondrocytes treated for 12 or 24 hours with conditioned medium from GM-cultured ASCs had reduced *sox9*, *acan*, and *col2 *mRNAs; reduced proliferation and proteoglycan synthesis; and increased apoptosis. ASC-conditioned medium also increased endothelial cell tube lengthening whereas conditioned medium from CM-cultured ASCs had no effect. Treating ASCs with CM reduced or abolished these deleterious effects while adding a neutralizing antibody for VEGF-A eliminated ASC-conditioned medium induced chondrocyte apoptosis and restored proteoglycan synthesis. FGF-2 also mitigated the deleterious effects VEGF-A had on chondrocyte apoptosis and phenotype. When GM-grown ASC pellets were implanted in 1 mm non-critical hyaline cartilage defects *in vivo*, cartilage regeneration was inhibited as evaluated by radiographic and equilibrium partitioning of an ionic contrast agent via microCT imaging. Histology revealed that defects with GM-cultured ASCs had no tissue ingrowth from the edges of the defect whereas empty defects and defects with CM-grown ASCs had similar amounts of neocartilage formation.

**Conclusions:**

ASCs must be treated to reduce the secretion of VEGF-A and other factors that inhibit cartilage regeneration, which can significantly influence how ASCs are used for repairing hyaline cartilage.

## Introduction

Adult stem cells, such as adipose stem cells (ASCs) are an emerging clinical option for treating tissue damage and diseases because of their accessibility and ability to differentiate into multiple cell lineages [1,2]. More recently, ASCs and other adult stem cells have been shown to secrete a wide range of trophic factors that can stimulate regeneration of tissues from multiple lineages [3]. As these cell therapies become more prevalent in different clinical procedures, their secretory profiles need to be more thoroughly investigated in terms of the role stem cell-secreted factors have in tissue regeneration and in possible side effects.

Hyaline cartilage is the most prevalent cartilaginous tissue found in the body, with adult stem cell therapies used to regenerate hyaline cartilage in the knee and trachea of humans [4,5] and to repair cartilage in the larynx, bronchial stump, and growth plate of animals in preclinical studies [6-8]. Unlike most other tissues, hyaline cartilage is an avascular and aneural tissue that has complex spatial variation in its mechanical properties and composition despite a relatively homogeneous cell population. Therefore, adult stem cell therapies used for repair of chondral defects must secrete factors that stimulate synthesis of specific proteoglycans and collagens without facilitating the infiltration of other tissues, especially blood vessel formation, or enabling hypertrophic differentiation of chondrocytes typical of endochondral ossification.

Although the secretory profile of ASCs for cartilage regeneration has been investigated [9], an in-depth study determining the role ASC-secreted factors have in chondrocyte proliferation, phenotype, and cartilage regeneration has yet to be conducted. Additionally, techniques such as microencapsulation and differentiation medium treatments have been used to improve the regenerative capacity for mesenchymal stem cells [10-12], but no study has investigated the effects these two parameters have on the secretion of trophic factors from ASCs. Therefore, the overall objective of this study was to determine the role ASC-secreted trophic factors have in cartilage regeneration. The effect of microencapsulation and chondrogenic medium treatment on angiogenic factor production was first determined. Then co-culture and conditioned media studies were conducted to determine how ASC-paracrine signaling and ASC-secreted factors affect chondrocytes. Finally, ASCs were implanted into a chondral defect in the xiphoid to determine the effect ASCs have on hyaline cartilage regeneration.

## Materials and methods

### Cell isolation

For each experiment, ASCs were isolated from inguinal fat pads of six 125-g male Sprague Dawley rats (Harlan Laboratories, Indianapolis, IN, USA) under a protocol approved by the Institutional Animal Care and Use Committee of the Georgia Institute of Technology as described in detail previously [13]. ASCs from the six animals were then combined and cultured in Lonza mesenchymal stem cell growth medium (GM) (Lonza, Walkersville, MD, USA). After one passage, these cells were positive for CD73 and CD271 and negative for CD45 [13]. Non-matching costochondral chondrocytes from the ribs of a different set of six 125-g male Sprague Dawley rats were isolated and combined as described previously [14]. Primary chondrocytes were cultured in DMEM containing 10% fetal bovine serum (FBS) and 50 μg/mL ascorbic acid (Invitrogen) until the fourth passage prior to experimental analysis. These cells continue to express type II collagen, aggrecan, and cartilage oligomeric matrix protein [14].

### Microencapsulation

**Figure 1:**
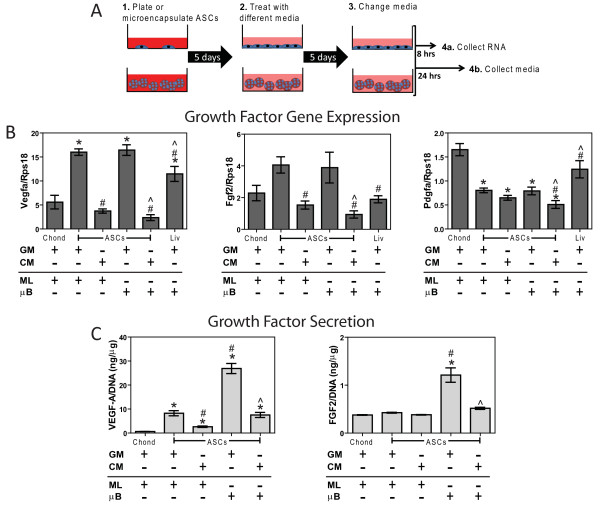
**Effects of microencapsulation and chondrogenic medium (CM) on angiogenic factor expression and secretion**. (**A**) Diagram of adipose stem cell (ASC) monolayer (ML) and microbead (μB) treatments with growth medium (GM) and CM prior to mRNA and media collections. (**B**) mRNA levels isolated after 5 days of culture and (**C**) growth factor production over the last 24 hours from ASCs, chondrocytes (chond), and liver cells (liv) (n = 6 ± SE). ^*^P < 0.05 vs. chond; ^#^*P *< 0.05 vs. ASCs; ^^^*P *< 0.05 vs. ASC microbeads (ASC + μB).

Once primary ASCs reached 90% confluence, cells were trypsinized and microencapsulated in 20 mg/mL low molecular weight (approximately 150 kDa) alginate (FMC BioPolymer, Sandvika, Norway) with a high mannuronate to guluronate ratio (40% guluronate) at a concentration of 25 × 10^6 ^cells/mL using a Nisco Encapsulator VAR V1 LIN-0043 (Nisco Engineering AG, Zurich, Switzerland) as previously described [15]. The microbeads were washed three times in GM prior to cell culture studies. First passage ASCs were also plated in 6-well plates (Figure 1A).

### ASC cell culture

Once first passage ASCs reached 90% confluence (Figure 1A), ASC monolayers and microbeads were then treated for 5 days with either GM or chondrogenic medium (CM) consisting of high-glucose DMEM with 1 mM sodium pyruvate (Mediatech, Manassas, VA, USA), 40 μg/mL proline (Sigma, St. Louis, MO, USA), 50 μg/mL ascorbate-2-phosphate (Sigma), 1% ITS+ (Sigma), 100 nM dexamethasone (Sigma), 10 ng/mL recombinant human transforming growth factor beta-1 (TGF-β1) (R&D Systems, Minneapolis, MN, USA) and 100 ng/mL recombinant human bone morphogenic protein 6 (BMP-6) (PeproTech, Rocky Hill, NJ, USA). Once media were changed on the fifth day, RNA was collected after 8 hours as described below while media and ASCs lysed in 0.05% Triton X-100 were collected after 24 hours. Fourth passage chondrocytes cultured in DMEM, 10% FBS, and 50 μg/mL ascorbic acid and Sprague Dawley-derived clone 9 liver cells (ATCC, Manassas, VA, USA) cultured in F12K medium and 10% FBS served as controls. All media contained 1% penicillin and streptomycin.

### Growth factor expression and production

Microbeads were un-cross-linked in 82.5 mM sodium citrate (Sigma), pelleted at 500 g for 10 minutes and washed two more times in sodium citrate to remove any residual alginate. TRIzol reagent (Invitrogen, Grand Island, NY, USA) was added to the resulting cell pellet, homogenized using a QIAshredder (QIAGEN, Valencia, CA, USA), and RNA was isolated using chloroform and an RNeasy Kit (Qiagen) as previously described [16]. Then 1 μg RNA was reverse-transcribed to cDNA using a High Capacity Reverse Transcription cDNA kit (Applied Biosystems, Carlsbad, CA, USA). Gene expressions were quantified as previously described [17]. Primers were designed using Beacon Designer software (Premier Biosoft, Palo Alto, CA, USA) and synthesized by Eurofins MWG Operon (Huntsville, AL, USA) unless otherwise noted (Table 1). Vascular endothelial growth factor (VEGF)-A and fibroblast growth factor (FGF)-2 production over the last 24 hours of culture was quantified using ELISA (R&D Systems) and normalized to DNA content measured with a Quant-iT PicoGreen kit (Invitrogen). Quantified mRNA levels are referred to by the name of the gene (for example, *fgf2*, *vegfa*, *runx2*) whereas quantified protein levels are referred to by the name of the growth factor (for example FGF-2, VEGF-A).

**Table 1:** Primer sequences

Gene	Direction	Sequence	Accession Number
*Acan*	Sense	GCT TCG CTG TCC TCA ATG C	NM_022190.1
	Antisense	AGG TGT CAC TTC CCA ACT ATC C	
*Col2*	Sense	CGAGTATGGAAGCGAAGG	NM_012929.1
	Antisense	GCTTCTTCTCCTTGCTCTTGC	
*Comp*	Sense	AGT GAC AGC GAT GGT GAT GG	NM_012834.1
	Antisense	TCC CCG TCC TGG TCT TGG	
*Fgf2*	Sense	Global Gene Sequence (Qiagen)	NM_019305.2
	Antisense		
*Pdgfa*	Sense	GAGGAGACGGATGTGAGG	NM_012801.1
	Antisense	ACGGAGGAGAACAAAGACC	
*Runx2*	Sense	TTGGACACCTTGGACGCTAATT	NM_053470.2
	Antisense	AGA GGC AGA AGT CAG AGG	
*Sox9*	Sense	GTG GGA GCG ACA ACT TTA CC	XM_003750950.1
	Antisense	ATC GGA GCG GAG GAG GAG	
*Vegfa*	Sense	GGACATCTTCCAGGAGTACC	NM_031836.2
	Antisense	CGTCTTGCTGAGGTAACC	

### Paracrine signaling

#### ASC-chondrocyte co-culture

**Figure 2:**
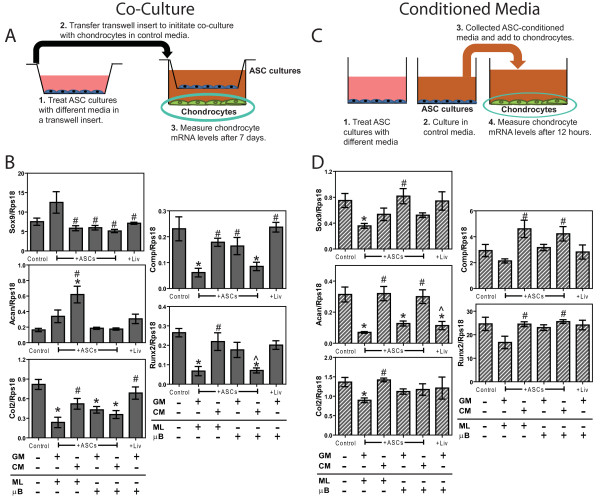
**Effects of adipose stem cell (ASC) co-culture and ASC-conditioned media on chondrocyte mRNA levels**. (**A**) Diagram of ASC co-culture and (**B**) mRNA levels of chondrocytes after 7 days. (**C**) Diagram of ASC-conditioned media treatment and (**D**) mRNA levels of chondrocytes after 12 hours. X-axis labels refer to treatments and culture type for ASCs and liver cells (Liv) prior to co-culture and conditioning, which include growth medium (GM), chondrogenic medium (CM), monolayer (ML), and microbead (μB) (n = 6 ± SE). ^*^P < 0.05 vs. control; ^#^*P *< 0.05 vs. ASCs, ^^^*P *< 0.05 vs. ASC microbeads (ASCs + μB).

To assess the effects that paracrine signaling between ASCs and chondrocytes have on chondrocyte phenotype, the two cell types were co-cultured in a trans-well system (Figure 2A). Initial studies determined the number of microbeads per insert needed to have the same cell number as ASC-confluent trans-well inserts. ASC monolayers and microbeads were treated with GM or with CM for 5 days in 0.2 μm high density cell culture inserts (BD Biosciences, Franklin Lakes, NJ, USA). ASC cultures and inserts were then washed in DMEM three times and added to wells with confluent chondrocytes. The two cell groups were then cultured in 4.5 mL DMEM + 10% FBS. After 7 days, RNA was isolated from the chondrocytes to quantify chondrogenic gene expression. Microencapsulated clone 9 liver cells served as a control.

#### Chondrocyte cultures treated with ASC-conditioned media

To assess the effects ASC-secreted factors have on them, chondrocytes were cultured in different ASC-conditioned media (Figure 2C) obtained from T-75 flasks. Initial studies determined the number of microbeads per T-75 needed to have the same cell number as ASC-confluent T-75 flasks. To obtain ASC-conditioned media, ASC monolayers and microbeads were treated with GM or CM for 5 days in T-75s. After the fifth day of treatment, ASC monolayers and microbeads were washed in DMEM three times and 10 mL DMEM + 10% FBS were added to each culture. After 24 hours, media containing the ASC-secreted factors were collected then immediately added to confluent chondrocyte cultures. After 12 hours in ASC-conditioned medium, RNA was isolated from chondrocytes to quantify chondrogenic gene expression. To assess chondrocyte phenotype, apoptosis, and proliferation, the following assays were performed after 24 hours of treatments with ASC-conditioned medium. These time points were selected based upon previous studies investigating the effects of different signaling molecules on chondrocytes [18-20]. Conditioned medium from microencapsulated clone 9 liver cells served as a control.

### Chondrocyte responses

#### Incorporation of [^3^H]-thymidine

DNA synthesis was assayed by measuring [^3^H]-thymidine incorporation as described previously [19]. Forty%-confluent chondrocytes were treated with DMEM + 1% FBS to induce quiescence. Four hours before harvest, [^3^H]-thymidine was added to a final concentration of 0.25 μCi/mL. Radioactivity in trichloroacetic acid-insoluble cell precipitates was measured by liquid scintillation spectroscopy.

#### Incorporation of [^35^S]-sulfate

Proteoglycan synthesis was assayed by measuring [^35^S]-sulfate incorporation as previously described [21]. The [^35^S]-sulfate was added to a final concentration of 18 μCi/mL for the final 4 hours of culture. Only [^35^S]-sulfate incorporation in the monolayer was measured since less than 15% of total radiolabeled proteoglycan production is secreted into the medium [20]. The [^35^S]-sulfate incorporation was normalized to protein content measured with a Pierce Macro BCA protein kit (ThermoScientific, Rockford, IL, USA).

#### Alkaline phosphatase activity

Alkaline phosphatase-specific activity in chondrocyte lysates was measured as a function of release of p-nitrophenol from p-nitrophenylphosphate as previously described [22] and normalized to protein content measured with a Pierce Macro BCA protein kit (ThermoScientific).

### Effects of ASCs on chondrocyte apoptosis

#### DNA fragmentation

To assess the effects of ASC-secreted factors on DNA fragmentation, confluent chondrocytes were pulsed with [^3^H]-thymidine for 4 hours prior to treatment with ASC-conditioned medium. Chondrocytes were lysed and centrifuged at 13,000 g for 15 minutes to separate intact DNA from fragmented DNA as previously described [18]. The amount of incorporated [^3^H]-thymidine was determined in each fraction to establish the total amount of fragmented DNA.

#### Caspase-3 activity

Caspase-3 activity was determined using a colorimetric CaspACE™ Assay System from Promega (Madison, WI, USA) following the manufacturer's protocol, 24 hours after ASC-conditioned media were added to confluent chondrocytes. Caspase-3 activity was normalized to total protein content measured with a Pierce 660 nm protein assay (ThermoScientific).

### Effect of secreted factors on angiogenic response

#### Fibrin gel assay

Angiogenic responses to different ASC-conditioned media were assessed with endothelial cells cultured in a fibrin gel assay as previously described [23]. Human aortic endothelial cells (HAECs) (Lonza) were plated in endothelial cell basal medium (EGM)-2 (Lonza) at 5 × 10^3 ^cells/well on fibrin gel and cultured at 37°C for 24 hours. At 24 hours, medium was removed, a second layer of fibrin was added on top, and ASC-conditioned media were added. After 12 hours, images were taken for morphometric analysis and total endothelial tube length was determined using Image Pro Plus.

#### Role of VEGF-A and FGF-2

To determine the effect exogenous VEGF-A and FGF-2 have on chondrocytes, 1 ng/mL and 20 ng/mL of recombinant human VEGF-A_165_, and recombinant human FGF-2 (R&D Systems), were added to monolayer cultures of fourth-passage chondrocytes. These concentrations were selected based upon a prior dose-response study on mesenchymal stem cells [24], and because measured VEGF-A concentrations among the different conditioned media groups ranged from 1 to 20 ng/mL. To determine the effect VEGF-A and FGF-2 secreted by ASCs have on chondrocytes, conditioned medium from GM-treated ASC monolayers was supplemented with 1 μg/mL goat anti rat IgG, 1 μg/mL goat anti-rat VEGF-A neutralizing antibody, or 1 μg/mL goat anti-rat FGF-2 neutralizing antibody (R&D Systems) and added to fourth-passage chondrocyte monolayers as determined by the manufacturer's protocol. After 24 hours, [^35^S]-sulfate incorporation, caspase-3 activity, and incorporation of [^3^H]-thymidine were measured as described above. DMEM + 10% FBS and conditioned medium from ASC monolayers treated with CM served as controls.

### Xiphoid defect *in vivo*

To assess if ASCs would inhibit cartilage regeneration, non-critically sized chondral defects were made in the xiphoids of 125-g male Sprague-Dawley rats as previously described [25]. The protocol was approved by the Institutional Animal Care and Use Committee of the Georgia Institute of Technology and each group was tested in seven rats. A full-thickness 1 mm cylindrical defect was made in the center of the xiphoid using a dermal biopsy punch (Mitex, Plainsboro, NJ, USA). This defect size was chosen because cartilage regeneration was previously observed after 35 days and the defect is large enough to contain cell pellets 1 × 10^6 ^ASCs in size [25]. ASC monolayers cultured in GM or CM were pelleted at 1 × 10^6 ^ASCs/pellet and implanted into the defect. Empty defects and autografts (excised cartilage is re-implanted) served as controls. To maintain groups in the defect and to serve as an adhesion barrier, SepraFilm® (Genzyme, Cambridge, MA, USA) was then placed on the dorsal and ventral sides of the defect. Xiphoids were excised 35 days post-surgery and examined as described below.

Radiographic imaging (Faxitron Bioptics, Lincolnshire, IL, USA) was performed in the coronal plane at a voltage of 22 mV and exposure time of 16 s to visualize soft tissue penetration as previously described [25]. Four observers with experience in examining radiographic images then scored the images blinded, for the presence of soft tissue penetration, with a score of 0 representing no healing, a score of 0.5 representing partial healing, and a score of 1 representing full healing. The average score each observer gave for each group was used for statistical analysis (n = 4).

Equilibrium partitioning of an ionic contrast agent via micro-computed tomography (EPIC-μCT, μCT40, SCANCO Medical, Brüttisellen, Switzerland) was used to visualize the distribution of proteoglycans within the xiphoid defects as previously described [25,26]. Xiphoids were incubated in 40% Hexabrix (Mallinckrodt, St. Louis, MO, USA) in PBS containing 1% proteinase inhibitor cocktail (CalBiochem, Darmstadt, Germany) overnight and scanned using pre-determined settings [25]. Low x-ray attenuation (green/yellow) of three-dimensional color images corresponded to regions of high proteoglycan concentration; no, or high x-ray attenuation (black and red/orange) indicated regions of low proteoglycan concentration. To determine cartilage volume, user-guided contours corresponding to a 1.1-mm cylinder around the defect were drawn and evaluated at a 100 to 250 threshold range. This contour size was chosen so all cartilage integrity, in-growth, and integration at the defect edge would be taken into account. Thus, all groups had positive cartilage volume since the evaluation volume was larger than the defect volume.

After scanning, samples were washed in PBS for four hours, fixed in 10% phosphate-buffered formalin for 48 hours, and embedded in paraffin. Serial sections, 7-μm thick, were stained with H&E to highlight cells and extracellular matrix on microscope images (DMLB; Leica, Nussloch, Germany).

### Statistical analysis

All *in vitro *experiments included six independent cultures per treatment group to ensure sufficient power to detect statistically significant differences. All *in vitro *experiments were conducted multiple times to validate the observations, but only data from a single representative experiment are shown and are expressed as means ± standard error (SE). A power analysis determined that seven samples per group were needed for the *in vivo *study based on results from a previous study [25]. Statistical analysis was conducted using analysis of variance (ANOVA) with the post hoc Tukey test (GraphPad Prism, La Jolla, CA, USA). Differences in means were considered to be statistically significant if the *P*-value was less than 0.05.

## Results

### Angiogenic growth factor production from ASCs

ASC monolayers and microbeads cultured in GM had 1.5 to 3.0 times higher levels of *fgf2 *and *vegfa *compared to chondrocytes while CM reduced both *vegfa *and *fgf2 *to levels similar to that of chondrocytes (Figure 1B). CM did not influence *pdgfa *in ASCs, which was half the mRNA level seen in chondrocytes. ASC monolayers and microbeads secreted 10 to 30 times more VEGF-A than chondrocytes, while CM reduced secretion by 3.0- to 3.5-fold (Figure 1C). FGF-2 secretion from ASC cultures and chondrocytes was very low compared to VEGF-A production.

### Effect of ASC paracrine signaling and secreted factors on chondrocyte gene expression

Chondrocytes co-cultured with ASC monolayers cultured in GM experienced 3.5- to 4.0-fold reductions in *col2*, *comp*, and *runx2 *compared to chondrocytes with no co-culture (Figure 2B). However, CM-treated ASC monolayers increased *acan *in chondrocytes and had no effect on *col2*, *comp*, and *runx2 *in chondrocytes compared to the control. GM-cultured ASC microbeads reduced *col2 *in chondrocytes but CM-treated ASC microbeads reduced *col2*, *comp*, and *runx2 *in chondrocytes compared to control. ASC co-cultures did not affect *sox-9 *and co-culture with clone 9 liver cell microbeads did not influence chondrocyte gene expression compared to the control.

Conditioned medium from GM-cultured ASC monolayers added to chondrocytes increased *sox9 *by 50%, *acan *by 78%, and *col2 *by 35% compared to control medium added to chondrocytes (Figure 2D). However, conditioned medium from CM-treated ASC monolayers had no effect on chondrogenic mRNA levels compared to control medium. Conditioned medium from GM-cultured ASC microbeads decreased *acan*, but conditioned medium from CM-treated ASC microbeads had no effect on chondrocyte mRNA levels. Clone 9 liver cell microbeads decreased *acan *in chondrocytes but had no effect on *sox9*, *col2*, *comp*, or *runx2*.

### Effect of ASC-secreted factors on proliferation, phenotype, and apoptosis

**Figure 3:**
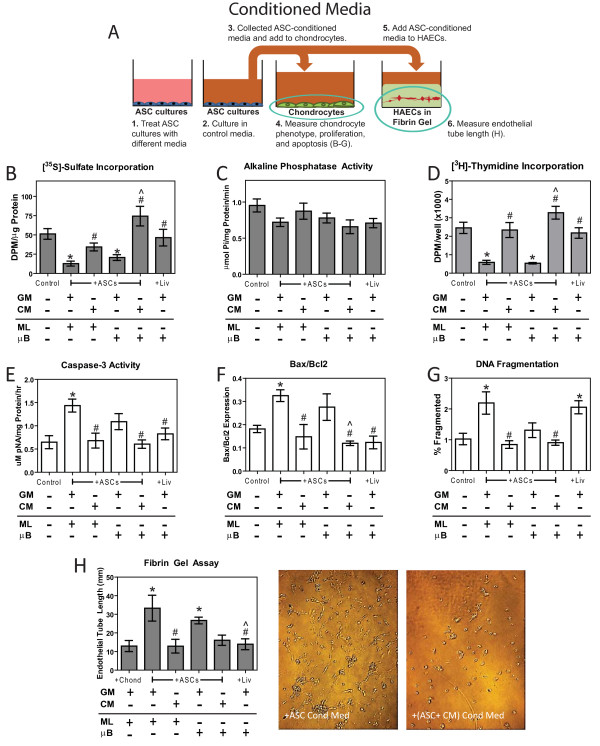
**Effects of adipose stem cell (ASC)-conditioned medium on chondrocyte phenotype, proliferation, apoptosis, and angiogensis**. (**A**) Diagram of ASC-conditioned media experiments, (**B**) [^35^S]-sulfate incorporation, (**C**) alkaline phosphatase activity, (**D**) [^3^H]-thymidine incorporation, (**E**) caspase-3 activity, (**F**) bax/bcl2 mRNA levels, (**G**) DNA fragmentation, and (**H**) endothelial tube length. All experiments were assayed after 24 hours of conditioned media treatment except for bax/bcl2 mRNA levels and endothelial tube length, which were measured after 12 hours of treatment. X-axis labels refer to treatments and culture type for chondrocytes (Chond), ASCs, and liver cells (Liv) prior to conditioning, which include growth medium (GM), chondrogenic medium (CM), monolayer (ML), and microbead (μB) (n = 6 ± SE). ^*^P < 0.05 vs. control; ^#^*P *< 0.05 vs. ASCs; ^^^*P *< 0.05 vs. ASC microbeads (ASCs + μB).

Conditioned media from GM-cultured ASC monolayer and microbeads decreased [^35^S]-sulfate incorporation by 75% and 60% respectively (Figure 3B), had no effect on alkaline phosphatase activity (Figure 3C), and decreased [^3^H]-thymidine incorporation by approximately 76% (Figure 3D) compared to chondrocytes treated with control medium. Treating ASC monolayers and microbeads with CM prior to conditioned media collection eliminated the deleterious effects ASC-secreted factors had on chondrocytes (Figure 3B, D). Liver microbead-conditioned medium did not affect [^3^H]-thymidine incorporation, [^35^S]-sulfate incorporation, or alkaline phosphatase activity (Figure 3B-D).

Conditioned medium from GM-cultured ASC monolayers increased caspase-3 activity 120%, bax/bcl expression 79%, and DNA fragmentation 114% compared to control medium (Figure 3E-G). Treating ASC monolayers with CM prior to collecting conditioned medium eliminated the apoptotic effects ASC-secreted factors had on chondrocytes. Conditioned medium from ASC microbeads cultured in GM did not increase apoptosis compared to control medium. Conditioned medium from liver microbeads had no effect on caspase-3 activity and bax/bcl, but did increase DNA fragmentation compared to the control (Figure 3E-G).

ASC monolayer and microbead-conditioned media increased endothelial tube length by 2.6- and 2.0-fold respectively (Figure 3G). Treating ASC monolayers and microbeads with CM prior to conditioned media collection eliminated this angiogenic response. Conditioned medium from liver microbeads had no effect on endothelial tube length.

### Effect of exogenous VEGF-A and FGF-2 on chondrocytes

**Figure 4:**
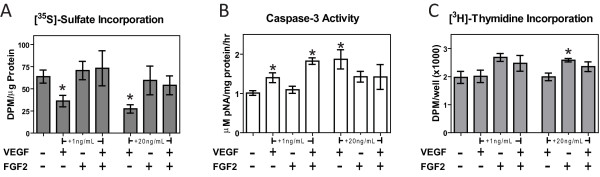
**Effects of exogenous vascular endothelial growth factor (VEGF)-A and fibroblast growth factor (FGF)-2 on chondrocytes**. (**A**) [^35^S]-sulfate incorporation of chondrocytes treated with recombinant human VEGF-A and FGF-2, (**B**) caspase-3 activity of chondrocytes treated with recombinant human VEGF-A and FGF-2, and (**C**) [^3^H]-thymidine incorporation of chondrocytes treated with recombinant human VEGF-A and FGF-2 after 24 hours of treatment (n = 6 ± SE). ^*^P < 0.05 vs. control; ^#^*P *< 0.05 vs. adipose stem cells (ASCs).

The incorporation of ^35^S-sulfate was reduced by approximately 30% and 50% with the addition of 1 ng/mL and 20 ng/mL of VEGF-A, respectively (Figure 4A). Adding FGF-2 at both 1 and 20 ng/mL alone had no effect on ^35^S-sulfate incorporation but did eliminate the inhibitory effect rhVEGF-A had on ^35^S-sulfate incorporation at both concentrations. Addition of VEGF-A at concentrations of 1 ng/mL and 20 ng/mL significantly increased caspase-3 activity by 32% and 84%, respectively (Figure 4B). Adding 1 ng/mL of FGF-2 had no effect on caspase-3 activity and did not reduce the apoptotic effect of VEGF-A compared to the control. However, 20 ng/mL FGF-2 eliminated the apoptotic effect of VEGF-A. VEGF-A and FGF-2 had a more limited effect on chondrocyte proliferation, as only the 20 ng/mL FGF-2 dosage increased [^3^H]-thymidine incorporation (Figure 4C).

### Effect of ASC-secreted VEGF-A and FGF-2 on chondrocytes

**Figure 5:**
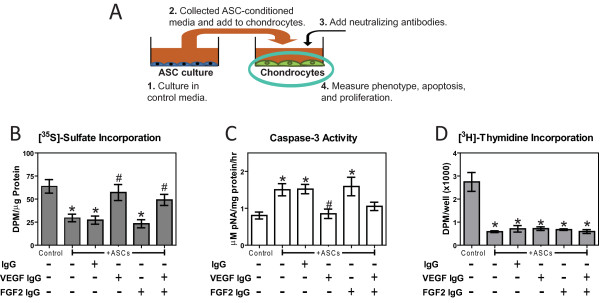
**Effects of adipose stem cell (ASC)-secreted vascular epithelial growth factor (VEGF)-A and fibroblast growth factor (FGF)-2 on chondrocytes**. (**A**) Schematic outlining chondrocytes treated with ASC-conditioned medium with VEGF-A and FGF-2 neutralizing antibodies and assayed for (**B**) [^35^S]-sulfate incorporation, (**C**) caspase-3 activity, and (**D**) [^3^H]-thymidine incorporation after 24 hours of treatment (n = 6 ± SE). ^*^P < 0.05 vs. control; ^#^*P *< 0.05 vs. ASCs.

ASC-conditioned medium with a goat-anti-rat IgG and FGF-2 neutralizing antibody decreased [^35^S]-sulfate incorporation by approximately 50% (Figure 5B), and increased caspase-3 activity by approximately 94% (Figure 5C) compared to control medium. Adding VEGF-A neutralizing antibody to ASC-conditioned medium eliminated both its deleterious effect on chondrocyte phenotype and its apoptotic effect. ASC-conditioned medium with or without VEGF-A or FGF-2 neutralizing antibodies all decreased [^3^H]-thymidine incorporation compared to control medium (Figure 5D). As previously observed, conditioned medium from ASC monolayers treated with CM had no effect on chondrocyte caspase-3 activity, [^35^S]-sulfate, and [^3^H]-thymidine incorporation compared to control medium (data not shown).

### Effect of ASCs in cartilage defects

**Figure 6:**
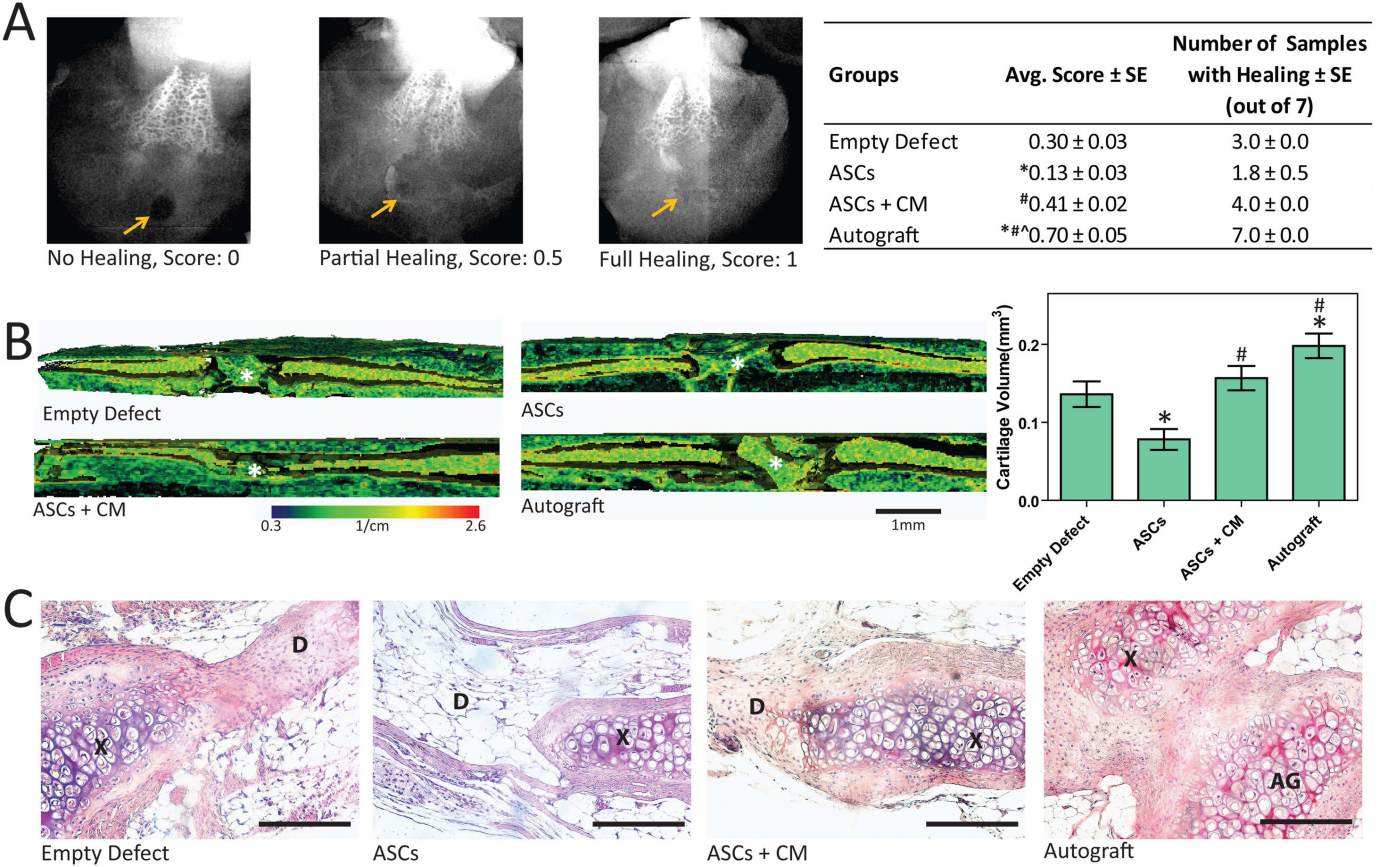
**Effects of adipose stem cells (ASCs) on cartilage regeneration**. (**A**) Radiographic scoring with arrows highlighting the defect (n = 4 blinded scoring averages ± SE). ^*^P < 0.05 vs. empty defect; ^#^*P *< 0.05 vs. ASCs; ^^^*P *< 0.05 vs. ASC + chondrogenic medium, CM). (**B**) Three-dimensional EPIC-micro computer tomography (μCT) images of xiphoids with ^*^marking the defect and calculated cartilage volume within defects (n = 7 ± SE). ^*^P < 0.05 vs. empty defect; ^#^*P *< 0.05 vs. ASCs. (**C**) Representative H&E staining. Bar represents 100 μm at 20 × magnification (D = defect, × = xiphoid, AG = autograft).

Scoring of radiographic images showed that all seven defects with autografts had partial or full healing and three of seven empty defects had partial or full healing; however, no more than two of seven defects with ASC pellets had partial healin, whereas four of seven defects with pellets of ASCs treated with CM had partial or full healing (Figure 6A). Scores for healing for the empty defects, defects with ASCs treated with CM, and defects with autografts were all higher than for defects with ASC pellets. Scores for defects with ASCs treated with CM were not statistically different from scores for the empty defects, and were lower than scores for defects with the autografts (Figure 6A).

Proteoglycan was visible in EPIC-μCT images of empty defects and defects with pellets of CM-treated ASCs but absent from defects with pellets of GM-cultured ASCs (Figure 6B). EPIC-μCT-calculated cartilage volume for defects with ASC pellets was significantly smaller than cartilage volume in both autografts and empty defects. Defects with CM-treated ASCs had more cartilage than defects with GM-cultured ASCs, but were not different from empty defects (Figure 6B).

In histological sections of defects with autografts, early-stage cartilage and infiltrating cells were starting to integrate the graft with the surrounding xiphoid (Figure 6C). Similar tissue deposition and cell infiltration was observed for empty defects and defects with ASCs treated with chondrogenic media. However, defects with ASCs were infiltrated with surrounding epithelial tissue instead and lacked the connective tissue that was observed in empty defects (Figure 6C).

## Discussion

This study is the first to definitively show that ASCs secrete angiogenic factors that are detrimental for chondrocytes and can prevent cartilage regeneration. Specifically, ASCs secrete a relatively large amount of VEGF-A, which can inhibit chondrocyte phenotype and lead to chondrocyte apoptosis. Additionally, ASCs secrete factors that negatively influence chondrocyte proliferation. Finally, ASCs prevent cartilage regeneration in a non-critical, hyaline cartilage defect *in vivo*. Despite these deleterious effects, treating ASCs with chondrogenic medium can reduce the expression and production of VEGF-A, eliminate the damaging effects ASC-secreted factors have on chondrocytes, and prevent the adverse effects ASCs have on regenerating cartilage *in vivo*.

In addition to chondrogenic medium, microencapsulation had a significant effect on VEGF-A and FGF-2 secretions. Specifically, microencapsulation had the opposite effect of chondrogenic medium and increased the secretion of these angiogenic factors. Hypoxia due to the high cell density within the microbead may have increased VEGF-A and FGF-2 secretions. Hypoxic conditions can increase VEGF-A and FGF-2 secretion from ASCs [27] since the genes for these angiogenic factors have hypoxia response elements [28,29]. Although we did not assay for hypoxic conditions within the microbead, increasing cell concentrations within hydrogels has been shown to increase oxygen tension and gradients [30]. Cell density may therefore be an important variable in controlling growth factor secretion from ASC microbeads.

Previous studies have extensively studied the secretory profiles of bone marrow-derived mesenchymal stem cells (MSCs) and ASCs, showing that they not only secrete several factors that can facilitate cartilage regeneration [9,31], but that they also secrete a large amount of angiogenic factors [32,33]. VEGF-A, an important initiator and mediator of angiogenesis [34,35], was secreted in large quantities by ASC monolayers and microbeads, decreased chondrocyte proteoglycan production, and induced chondrocyte apoptosis in the current study. Although VEGF-A typically elicits anabolic responses from most cell types, this growth factor has catabolic effects on chondrocytes and has been shown to induce matrix metalloproteinase expression in these cells [36]. Although VEGF-A was not responsible for reduced chondrocyte proliferation due to ASC-secreted factors, ASCs also secrete pro-inflammatory factors IL-6, 8, and 11, and TNFα [37], which may have inhibited chondrocyte proliferation.

Although VEGF-A alone did not prevent chondrocyte proliferation, it mitigated the ability of FGF-2 to stimulate chondrocyte proliferation at a high concentration. Additionally, FGF-2 restored proteoglycan production in chondrocytes treated with VEGF-A, and high concentrations of FGF-2 eliminated the apoptotic effect VEGF-A had on chondrocytes. While both FGF-2 and VEGF-A are important facilitators for angiogenesis [38,39] and their corresponding mRNA levels in ASCs experienced similar decreases when treated with chondrogenic medium, ASCs secreted at least 10-fold less FGF-2 than VEGF-A. Furthermore, unlike VEGF-A, FGF-2 has previously been shown to enhance the chondrogenic potential of different stem cell sources [40,41], as well as improve cartilage regeneration [42,43]. The combination of high VEGF-A secretion and low FGF-2 secretion may be the main reason why ASC-secreted factors have deleterious effects on chondrocytes and subsequent cartilage regeneration.

VEGF enhances catabolic pathways in chondrocytes, and VEGF signaling has been associated with osteophyte formation and progression of osteoarthritis in articular cartilage [44-46]. Moreover, blocking VEGF signaling improves the chondrogenic potential and regenerative capacity of muscle-derived stem cells [47,48]. Additionally, VEGF-A directs cartilage vascularization in hyaline cartilage and hypertrophic chondrocyte absorption that leads to subsequent ossification [49,50]. Although chondrocytes exposed to ASC-secreted factors did not have increased mRNA levels corresponding to *RUNX2 *or increased alkaline phosphatase activity, and cartilage defects with ASC pellets did not have signs of vascularization, hypertrophic differentiation, or bone formation, *in vitro *conditioned media studies longer than 24 hours or *in vivo *studies longer than 35 days may have revealed these effects. Additionally, ASCs may secrete trophic factors that prevent hypertrophic differentiation.

In addition to affecting hypertrophic differentiation, the length of exposure to ASC-secreted factors and the time points measured appear to be important variables in regulating chondrocyte phenotype. Twelve hour exposure to ASC-conditioned medium appeared to have the most significant effects on mRNA levels corresponding to *SOX9 *and aggrecan, which are earlier stage markers of chondrogenesis, whereas 7 days of ASC co-culture exerted the greatest effects on mRNA levels corresponding to *RUNX2*, a later stage chondrogenic marker [51]. Because these unique phenotypic expression profiles for the different stages of chondrogenesis, the time points measured for the co-cultures, conditioned medium, and subsequent studies may have affected the findings on chondrocyte phenotypic expression, proliferation, proteoglycan synthesis, and apoptosis. However, these time points are consistent with previous studies investigating the effects different signaling molecules have on chondrocytes [18,19,21]. Additionally, 7-day co-culture with ASCs treated with chondrogenic medium increased aggrecan expression in chondrocytes, and this increase was not observed in the conditioned media study. Although the different time points between the two studies may explain this discrepancy, the interplay between chondrocyte and ASC signaling may also have a contribution.

Several studies have investigated the effects chondrocyte and stem cell co-cultures and chondrocyte-secreted factors have on stem cell differentiation [47,52,53], but few have provided insight into the effects stem cell-mediated paracrine signaling has on chondrocytes. Bian *et al*. showed that co-culturing chondrocytes with MSCs within the same gel reduced hypertrophy and increased compressive moduli, but it appeared that these improvements were independent of long-range MSC-paracrine signaling [54]. Additionally, results from Hildner *et al*. suggested that co-culturing ASCs and chondrocytes in the same matrix moderately increased *SOX9*, aggrecan, and cartilage oligomeric protein mRNA expression when normalized to the initial percentage of chondrocytes, but the findings provided little insight into the role of ASC-secreted factors [55]. Although chondrogenic treatment that included both TGF-β1 and BMP-6 eliminated the deleterious effects that ASC-secreted factors and ASCs had on chondrocytes and cartilage regeneration respectively, ASCs treated with chondrogenic medium did not secrete factors that improved the ability of chondrocytes to produce cartilaginous extracellular matrix *in vitro*. This absence of improvement over baseline may be due to the short 5-day chondrogenic medium-treatment time as several studies have shown optimal ASC and stem cell chondrogenesis to require at least 2 to 6 weeks of chondrogenic treatment [56,57]. However, these studies did not investigate the effects that varying the temporal application of chondrogenic medium had on trophic factor production. Additionally, assessing cartilage regeneration at longer time points post-operatively may provide better insight into the therapeutic benefit of this group.

## Conclusions

This study showed that ASCs can inhibit cartilage regeneration within a focal hyaline cartilage defect *in vivo *after 35 days. *In vitro*, ASC monolayers or microbeads cultured in growth medium secreted a large amount of VEGF-A, which caused chondrocyte apoptosis and reduced proteoglycan synthesis after 24 hours of conditioned media treatments. Additionally, these ASCs secreted factors that reduced chondrogenic gene expression and proliferation after 12 and 24 hours of conditioned media treatments respectively. Treating ASCs with chondrogenic medium for 5 days reduced the secretion of VEGF-A, significantly reduced the deleterious effects ASC-secreted factors have on chondrocytes, and eliminated the inhibitory effect ASCs have on cartilage regeneration. Blocking VEGF-A in ASC-conditioned medium eliminated the deleterious effects ASC-secreted factors had on chondrocyte phenotype and apoptosis after 24 hours. Adding a high concentration of FGF-2 eliminated the apoptotic effect VEGF-A had on chondrocytes, and adding both low and high concentrations of FGF-2 eliminated the detrimental effects VEGF-A had on chondrocyte phenotype after 24 hours. These results have significant implications on how ASCs and possibly other stem cell therapies are used for repairing cartilage. Specifically, these therapies must be pre-treated or modified to reduce the inhibitory effects of VEGF-A and other secreted factors on cartilage regeneration.

## Abbreviations

*ACAN*: aggrecan; ANOVA: analysis of variance; ASCs: adipose stem cells; BMP-6: bone morphogenetic protein-6; cDNA: complementary deoxyribonucleic acid; CM: chondrogenic medium; *COL2*: type-II collagen; *COMP*: cartilage oligomeric matrix protein; ELISA: enzyme-linked immunosorbent assay; EPIC-μCT: equilibrium partitioning of an ionic contrast agent via micro-computed tomography; FBS: fetal bovine serum; FGF-2: fibroblast growth factor-2; GM: growth medium; IL: interleukin; MSCs: mesenchymal stem cells; PBS: phosphate-buffered saline; RPS18: ribosomal protein S18; *RUNX2*: runt-related transcription factor 2; SE: standard error; *SOX9*: sex determining region Y-box containing gene 9; TGF-β1: transforming growth factor-beta 1; TNF: tumor necrosis factor; VEGF-A: vascular endothelial growth factor-A.

## Competing interests

CSD Lee, BD Boyan, and Z Schwartz are co-inventors of the cell culture and microbead technologies described in this research, which is licensed to SpherIngenics, Inc. BD Boyan and Z Schwartz are co-founders and own stock in SpherIngenics, Inc. OA Burnsed, V Raghuram, and J Kalisvaart have no competing interests.

## Authors' contributions

All authors have read and approved the manuscript for publication. Authors have contributed to the conception and design (CSDL, BDB, ZS), collection and assembly of data (CSDL, OAB, VR, JK), data analysis and interpretation (CSDL, OAB, VR, JK), writing (CSDL, ZS), editing (CSDL, BDB), financial and administrative support (BDB), and final approval (BDB, ZS) associated with this manuscript.
